# Changes in the Lipid Asset of HIV/HCV Patients after a Successful Course of Direct-Acting Antivirals

**DOI:** 10.3390/jcm13133865

**Published:** 2024-06-30

**Authors:** Anna Maria Spera, Valeria Conti, Graziamaria Corbi, Tiziana Ascione, Michele Ciccarelli, Alfonso Masullo, Gianluigi Franci, Pasquale Pagliano

**Affiliations:** 1 Unit of Infectious Diseases, Department of Medicine, Surgery and Dentistry, “Scuola Medica Salernitana”, University of Salerno, 84081 Baronissi, Italy; aspera@unisa.it (A.M.S.); ppagliano@unisa.it (P.P.); 2 UOC Clinica Infettivologica AOU San Giovanni di Dio e Ruggi d’Aragona, 84131 Salerno, Italy; 3 Unit of Pharmacology, Department of Medicine, Surgery and Dentistry, “Scuola Medica Salernitana”, University of Salerno, 84081 Baronissi, Italy; vconti@unisa.it; 4 Department of Translational Medical Sciences, University of Naples Federico II, 80131 Naples, Italy; 5 Service of Infectious Diseases AORN A Cardarelli, 80131 Naples, Italy; tizianascione@hotmail.com; 6 Unit of Cardiology, Department of Medicine, Surgery and Dentistry, “Scuola Medica Salernitana”, University of Salerno, 84081 Baronissi, Italy; mciccarelli@unisa.it; 7 UOC Malattie Infettive AOU San Giovanni di Dio e Ruggi d’Aragona, 84131 Salerno, Italy; alfonso.masullo@sangiovannieruggi.it; 8 Unit of Microbiology, Department of Medicine, Surgery and Dentistry “Scuola Medica Salernitana”, University of Salerno, 84081 Baronissi, Italy; gfranci@unisa.it

**Keywords:** HIV, HCV, HAART, DAA, co-infection, dyslipidemia

## Abstract

**Background:** Highly Active Antiretroviral Therapy (HAART) for HIV infection and Direct-Acting Antivirals (DAA) for HCV infection currently represent the main treatment options for HIV/HCV co-infected patients. However, HAART has been associated with increased lipids. This study aimed to evaluate lipid profile changes after the DAA cycle in HIV/HCV co-infected patients undergoing HAART/DAA therapy. **Methods:** A prospective, longitudinal, observational study among HIV/HCV co-infected patients undergoing HAART/DAA treatment was conducted at the Infectious Diseases Unit of the University Hospital of Salerno. Inclusion criteria were age > 18 years, written informed consent, completion of the DAA cycle, and virologic suppression on HAART. Changes in the lipid profile were analyzed from baseline during and after DAA therapy at 12, 24, and 48 weeks after the sustained virologic response (SVR). A t-test was used to compare continuous variables. An analysis of variance was performed for each antiretroviral drug and genotype. **Results:** Fifty-four HIV/HCV patients (men/women n. 34/20 [68/32%], median age 56 years), all naïve to HCV therapy, were enrolled. HCV infection was caused by genotype 1 in 55% of cases and by genotype 3 in 29%. An increase in total cholesterol was recorded after the DAA treatment (from 165.03 ± 46.5 to 184.7 ± 44.9 mg/dL, *p* < 0.0001), after 12, 24, and 48 weeks, and in LDL-C at 24 weeks follow-up (at baseline 86.7 ± 34 mg/dL to 103.4 ± 41.38 mg/dL, *p* < 0.0001). **Conclusions:** Changes in the lipid profile after combined DAA/HAART treatment represent an important prognostic index. Further evaluation of cardiovascular-associated risk is necessary to implement appropriate prevention strategies.

## 1. Introduction

The World Health Organization (WHO) reports that approximately 2.3 million of the estimated 37.7 million people living with HIV (PLWH) are also co-infected with the Hepatitis C Virus (HCV). This population constitutes a major problem in terms of liver disease or HIV management [1]. Based on epidemiological and clinical data, it can be assumed that co-infected PLWH have a worse outcome with HCV liver disease compared to HCV patients who are HIV seronegative because HIV can alter the efficiency of the immune system, potentially leading to a less effective immune response against the HCV infection. Additionally, as an HIV infection facilitates the progression of liver fibrosis, it has been shown that the administration of highly active antiretroviral therapy (HAART) delays the progression of fibrosis, thus reducing the progression to end-stage liver disease in co-infected patients [2,3]. On the other hand, HCV infection affects the outcome of HIV patients by slowing down the immune reconstitution process during HAART. Moreover, DAA therapy for HCV can mobilize the HIV viral reservoir from tissues, increasing the HIV replication load [4,5]. Dyslipidemia is one of the most clinically relevant adverse drug reactions associated with the drugs belonging to the HAART regimen [6]. Several prospective studies have found a significant increase in low-density lipoprotein cholesterol (LDL)-C among HIV+ patients after starting HAART, resulting in an increased cardiovascular risk [7,8].

Protease inhibitors are the most involved, but other components of the HAART regi-men may be responsible for the altered lipid balance [9,10]. Moreover, as there is a clear association between HIV infection and incident atrial fibrillation (AF), a therapeutic approach for the treatment and prevention of AF recurrence among PLWH is required. For this reason, La Fazia et al. conducted a prospective interventional study of 1438 consecutive patients with HIV (cases) and 1407 seronegative ones (controls) that were propensity score matched [11]. After a 1-year follow-up, these authors found a higher rate of arrhythmia recurrence in the mid- and long-term for HIV+ AF patients [11].

HCV infection has been found to cause changes in the serum lipid profile by interfering with the metabolic pathway of very low-density lipoproteins (VLDL) during the assembly and secretion process within hepatocytes. In particular, HCV infection increases hepatic lipogenesis and reduces lipid oxidation and efflux from hepatocytes [12,13]. While the association between HAART and dyslipidemia has been largely elucidated in HIV patients, there are a lack of data on changes in the lipid profile of HIV/HCV co-infected patients receiving combined HAART/DAA treatment. In particular, how HCV replication affects the lipid profile is still debated, considering what happens after virus clearance by effective DAA treatment [14]. This study aimed to evaluate the changes in lipid profile occurring after DAA treatment and the resulting changes in cardiovascular risk in patients with HIV/HCV co-infection.

## 2. Materials and Methods

This is a prospective, longitudinal, observational study examining HIV/HCV-positive patients referred to the Infectious Diseases Unit of the University Hospital “San Giovanni di Dio and Ruggi d’Aragona”, a tertiary-care center where HIV patients from a 1-million-inhabitant-area converge.

### 2.1. Study Design

PLWH who had received HAART for at least 6 months were included in this study from 2017 to 2022 if HCV-Ab positive and with a positive test for HCV-RNA. Inclusion criteria were: (1) established diagnosis of HCV chronic hepatitis as assessed by detectable HCV-RNA; (2) co-presence of HIV infection in PLWH under HAART with HIV RNA undetectable (<40 copies/mL) and lymphocytes CD4 > 200 cell/mL for at least 6 months; (3) age > 18 years; (4) treatment with DAA based on HCV genotype; and (5) follow-up until 48 weeks after SVR. Exclusion criteria were: (1) co-infection with HBV; (2) no response or relapse after receiving DAA or other antiviral therapy for HCV; (3) concomitant diagnosis of cancer outside the liver within 5 years of admission; or (4) dyslipidemia on cholesterol-lowering treatment.

Patients were managed and underwent DAA treatment as follows:

- A case report form was completed for each patient at baseline that included socio-demographic and epidemiological characteristics (age, sex, HCV genotype, risk factors for HIV and HCV) at baseline, as well as the degree of liver stiffness assessed by liver elastosonography, the HAART in use at the time of enrollment (and the possible switch before starting the DAA therapy), the immune profile (lymphocyte subpopulations), and HIV-RNA and HCV-RNA assays at baseline. Other laboratory investigations, including blood sugar level, parameters of the liver function (AST, ALT, GGT, total bilirubin), renal function parameters (blood urea, creatinine), blood parameters (INR, hemoglobinemia, platelet, and leukocyte counts), and a lipid profile (total cholesterol, high-density lipoprotein [HDL], LDL, and triglycerides) were reported for each patient.
- Laboratory and virologic examinations were scheduled from baseline until completion of DAA treatment and at 12, 24, and 48-week follow-ups.

Changes in total cholesterol, LDL-C, HDL-C, and triglycerides during and after the DAA therapy were analyzed. The effects of specific antiretrovirals (tenofovir alafenamide fumarate, tenofovir disoproxil fumarate, abacavir, integrase inhibitors, and protease inhibitors) on lipid profile changes during the DAA therapy and the follow-up were examined. Any possible correlation between HCV genotype and changes in the lipid profile during the DAA therapy was analyzed. Cardiovascular risk was evaluated at baseline and after SVR using the Score2 risk prediction algorithm [15]. This study followed the Declaration of Helsinki and its amendments, without interfering with normal clinical practice, and was approved by the local ethics committee (n.30_r.p.s.o./2020).

### 2.2. Statistical Analysis

The descriptive statistics (expressed as mean, median, and standard deviation, or as a percentage and interquartile range for categorical variables) of the laboratory values were calculated at the time of enrollment, at the end of therapy, and at 12, 24, and 48 weeks follow-up after reaching the SVR. A t-test was used to verify whether the differences between the values found were significant; *p*-values below 0.05 were considered to be significant. Multiple regression analysis was performed for each antiretroviral drug to assess the possible causal effect of each drug on the lipid profile. The same statistical analysis was performed for each genotype to evaluate the possible causal effect of each drug on the lipid profile.

## 3. Results

The study included 54 patients with HIV/HCV co-infection receiving HAART/DAA combination therapy. The adherence to the HAART was assessed and maintained at each medical check-up through a self-administered survey. An SVR after the DAA therapy was achieved in 100% of the enrolled patients. The characteristics of the participants are summarized in Table 1.

The mean age of the patients was 54.2 years (median 56 years, SD ± 8.3) and 34 (68%) patients were male. Eighteen (31%) patients had liver cirrhosis defined by clinical criteria or based on a liver stiffness > 13 kPa, as assessed by elastosonography. The scheme of the HAART protocols administered at the time of starting the DAA therapy is summarized in Table 1.

**Figure 1:**
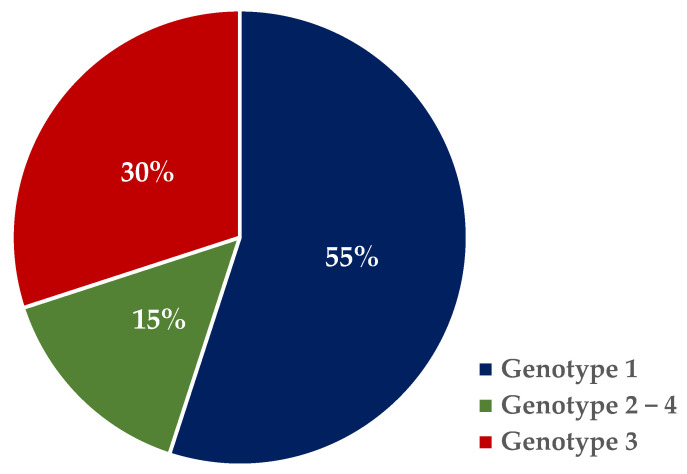
HCV genotypes of participants enrolled in the study.

Figure 1 shows the distribution of the HCV genotypes in the study population. Genotype 1a (*n* = 30, 55%) and genotype 3 (*n* = 16, 29%) were reported with the highest frequency (Figure 1). All participants were naïve to HCV therapy. The Sofosbuvir/Velpatasvir combination (18 cases, 33%) was the treatment administered with the highest frequency.

**Figure 2:**
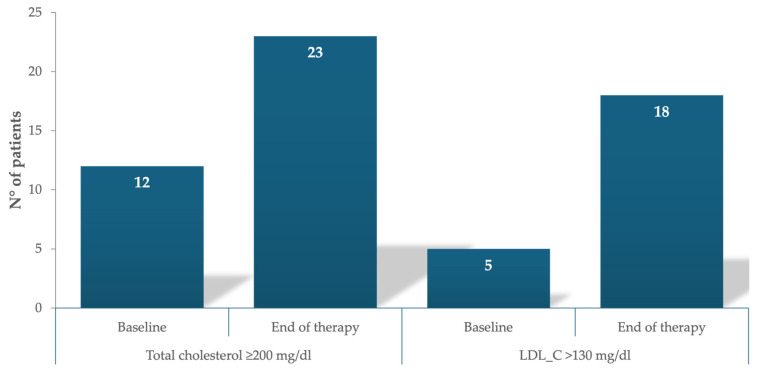
Change in the number of patients with abnormal lipid profiles (total cholesterol ≥ 200 mg/dL and LDL cholesterol > 130 mg/dL) at baseline and at the end of therapy.

After HCV eradication, liver function improved in all cases. The mean serum values of total cholesterol showed a significant increase at the end of the DAA treatment (165.03 ± 46.5 vs. 184.7 ± 44.9 mg/dL; *p* < 0.0001), while HDL-C and triglyceride levels remained unchanged. This increase remained stable during the follow-up, as reported in Table 2 and Figure 2.

Changes in the HAART regimen were made in 13 patients, without involving TDF or TAF, before they started the DAA therapy. However, no statistically significant changes in their lipid profiles were reported.

The mean value of LDL-C at 24 weeks after SVR was higher than the baseline value (103.4 ± 41.38 vs. 86.7 ± 34 mg/dL, *p* < 0.0001) (Table 2).

Multiple regression analysis demonstrated that the use of DRV/r as part of the HAART during HCV eradication with DAA treatment was associated with increased total cholesterol (*p* < 0.001), which persisted even after adjustments for the length of therapy since the HIV diagnosis and the previous use of other protease inhibitors. Moreover, participants receiving abacavir also showed a significant increase in total cholesterol (*p* < 0.001) after treatment with DAA. Patients exposed to TDF showed an increase in HDL levels after adjusting for potential confounders.

No other statistically significant changes were reported with other antivirals. Furthermore, it was observed that the regimens containing sofosbuvir did not affect the changes in lipid metabolism after the SVR was achieved. Finally, it was shown that the effect of HCV eradication on lipid metabolism varies according to the HCV genotype of the participants. The increase in total cholesterol values following DAA treatment was greater in patients with the HCV genotype 3a than in the participants with other HCV genotypes.

The overall cardiovascular risk was assessed at baseline and after the SVR using the SCORE2 risk prediction algorithm to estimate the 10-year risk of cardiovascular disease in our Italian cohort of both sexes, aged 40–69 years, without previous CVD or diabetes, considered to be at “moderate risk” for CVD according to the WHO [15]. Four patients were not eligible and thus are not included in the risk evaluation. Competing risk-adjusted models such as age, smoking status, systolic blood pressure, total, and HDL cholesterol were analyzed. A significant increase in CVD risk (SCORE2 7.4 vs. 7.1) was reported after the SVR (*p* = 0.004).

## 4. Discussion

This study investigated the impact of DAA therapy on lipid metabolism in patients with HIV/HCV co-infection who were already on the HAART regimen. The results indicate that HIV/HCV co-infected patients who achieve an SVR with DAA treatment experience a significant increase in serum levels of total cholesterol and LDL-C, without changes in serum levels of HDL-C and triglycerides. These results are in line with previous studies in HCV-positive individuals without HIV infection, in which similar changes in lipid profiles were observed following effective DAA treatment [16,17,18,19].

Baseline findings in our study population indicate that HIV/HCV co-infected patients have low serum total cholesterol levels, as well as both LDL-C and HDL-C levels. Several factors contribute to this phenomenon. Firstly, several critical steps of the HCV life cycle are closely linked to lipid metabolism, as the virus uses lipids to produce HCV particles in the bloodstream, lowering total cholesterol and LDL-C levels. Secondly, HCV-induced liver damage can impair hepatic synthesis and lipoprotein metabolism. Finally, HCV switches the production of VLDL to lipoviral particles (LVP) and thus alters lipid homeostasis by promoting intra-hepatic triglyceride accumulation [20]. These observations suggest that HIV individuals co-infected with HCV may present a unique lipid profile different from that of HIV-mono-infected patients. This specific lipid profile may potentially confer a lower cardiovascular risk than in HIV-mono-infected individuals. The results of our study provide valuable insights into the impact of successful anti-HCV therapy on lipid profiles in PLWH and co-infected with HCV. The study showed a 100% cure rate for HCV infection, with a significant effect on the lipid profile in terms of increased total cholesterol and low-density lipoproteins and with no significant impact on high-density lipoproteins. Once HCV replication is effectively inhibited by anti-HCV therapy in PLWH, underlying alterations in lipid metabolism—previously undetected—become apparent. This suggests that lipids may accumulate in the vascular intima, as observed in HIV-positive patients without HCV infection [21]. In this context, cardiovascular risk may worsen due to the deleterious effect of HIV and antiviral therapy on the endothelium, which is further exacerbated by alterations in the lipid profile, as demonstrated by the increase in the Score2 risk prediction algorithm. On the basis of these considerations, lipid profile monitoring, diet modification, and the use of cholesterol-lowering drugs should be considered to reduce the cardiovascular risk, where appropriate, when a successful DAA therapy has been administered to HCV/HIV co-infected patients. With regard to the aforementioned increased risk of cardiovascular events among PLWH, it should be noted that HIV/HCV co-infected subjects experience an apparent “PCSK9-Lipid paradox”, meaning that serum LDL-C remains low despite a high concentration of Proprotein Convertase Subtilisin Kexin 9 (PCSK9), in parallel with proatherogenic inflammation marker elevation, which usually reduces serum LDL-C. Although PCSK9 inhibition may be a potentially attractive therapy for dyslipidemia treatment, this paradox makes it difficult to determine the efficacy of targeted PCSK9 inhibition in HIV/HCV co-infected patients [22].

An important result of this study concerns the impact of HCV genotype 3 clearance on the lipid profile of HIV/HCV co-infected subjects. Younossi et al. demonstrated that the clearance of HCV genotype 3 in mono-infected patients resulted in a significant increase in total cholesterol, LDL-C, HDL-C, distal sterol metabolites, and apoB, as well as a significant decrease in apoE. These changes were confirmed during the follow-up, indicating that HCV suppression by antivirals in genotype 3 subjects with reduced circulating lipids levels can restore distal sterol metabolism [23,24]. These findings are consistent with the lipid profile changes observed in the HIV-positive subjects with HCV genotype 3 coinfection who were enrolled in our study, indicating that the mechanisms of lipid metabolism in the HIV/HCV co-infected population do not differ from the HCV mono-infected patient population. This represents a novelty, especially considering how this subclass is underrepresented in clinical trials.

In addition, this study addressed the possible impact of specific antiretroviral regimens on lipid changes in subjects before and after HCV eradication. It was observed that subjects exposed to darunavir had an increase in total cholesterol post-SVR that persisted even after adjustments for the time interval since HCV diagnosis and for the previous use of other protease inhibitors [24]. Furthermore, an increase in HDL-C levels was recorded among subjects exposed to TDF, highlighting the influence of this drug on the lipid profile. All these changes are expected, considering other factors. Aging with an HIV/HCV co-infection is a risk factor for developing neurocognitive impairment because the chronic inflammation triggered by these viruses can promote neurodegeneration through the acceleration of cellular aging and trigger neuropathogenesis through the accumulation of α-synuclein, amyloid β, or tau in the co-infection setting [25]. Thus, treating HCV infection among HIV co-infected individuals appears mandatory to reduce the risk of developing a neurocognitive impairment but can worsen the lipid profile, increasing the cerebrovascular degenerative process. Therefore, a comprehensive approach addressing both viral infections and lipid profile changes should be adopted in these patients to reduce the risks of both virus-induced neurodegenerative processes and to manage cardiovascular risk [26]. It should be noted that these patients have a high rate of chronic hepatitis, which progresses more rapidly to end-stage liver disease (cirrhosis) and hepatocellular carcinoma (HCC) if left untreated.

In this regard, HCV treatment plays a key role in limiting the progression of liver disease and reducing the risk of HCC development in mono- and co-infected individuals, especially when used at an early stage of fibrosis, thereby reducing liver disease mortality and morbidity [27].

HCV eradication in patients co-infected with HIV has a favorable impact on the progression to liver cirrhosis and HCC.

### Limitations and Strengths

The main limitation of the present study is the small sample size. However, this study investigated the impact of DAA therapy on lipid metabolism in patients with an HIV/HCV co-infection undergoing HAART/DAA treatment, which resulted in an increase of SCORE2, demonstrating that these changes affect the 10-year risk of cardiovascular diseases. Surely, other studies including a bigger sample size are needed to confirm our results.

## 5. Conclusions

In conclusion, our study highlights that HIV/HCV co-infected patients undergo changes in lipid profile after a successful DAA treatment. Antivirals such as tenofovir and Darunavir can influence these changes and should be considered when planning anti-HIV treatment. Appropriate monitoring and additional treatment strategies (e.g., the use of statins and antiplatelet drugs) are required due to the new onset lipid profile alterations and to avoid a further increase in cardiovascular risk associated with HIV infection and HAART.

## Figures and Tables

**Table 1:** Baseline characteristics of the enrolled HIV/HCV subjects.

**Sample size,** *n*	54
**Recruitment period,** *range of years*	2017–2022
**Middle age,** *years (median* ± *SD)*	54.2 (56 ± 8.3)
**Patients in HAART,** *n* (%)	54 (100)
**Patients with HIV-RNA < 40 copies/mL,** *n* (%)	54 (100)
**CD4,** *mean ±SD*	958 ± 256
**Patients with CD4 > 500/mm^3^,** *n* (%)	15 (27.7)
**Years since HIV diagnosis,** *ys (IQR)*	18 (14–26)
**HAART,** *n* (%)	
TDF	22 (40.7)
TAF	3 (5.5)
ABC	8 (14.8)
DRV/r,	11 (20.3)
ATV/r	7 (12.9)
Raltegravir	8 (14.8)
**CAH HCV,** *n* (%)	37 (68)
**Cirrhosis,** *n* (%)	17 (31)
**Naïve to DAA,** *n* (%)	54 (100)
**HCV-RNA, log_10_ copies/mL,** *mean value*	3.38
**Genotype 1,** *n* (%)	30 (55) → 22a, 7b, 1ab
**Genotype 2,** *n* (%)	2 (3)
**Genotype 3,** *n* (%)	16 (29)
**Genotype 4,** *n* (%)	6 (11)
**DAA,** *n* (%)	
Ombitasvir/Paritaprevir/Ritonavir + Dasabuvir and Ribavirin	3 (5.6)
Daclatasvir + Sofosbuvir	11 (20.4)
Elbasvir + Grazoprevir	2(3.7)
Sofosbuvir + Velpatasvir	18 (33)
Glecaprevir + Pibrentasvir	3(5.6)
Ledipasvir + Sofosbuvir	13 (24.1)
Simeprevir	2(3.7)
Sofosbuvir + Ribavirin	2(3.7)
**Pretreatment total cholesterol** (<200 mg/dL), *n* (%)	42 (78)
**Pretreatment total cholesterol** (>200 mg/dL), *n* (%)	12 (22)

HAART, Highly Active Antiretroviral Therapy; HIV, human immunodeficiency virus; TDF, tenofovir disoproxil fumarate; TAF, tenofovir alafenamide fumarate, ABC, abacavir; DRV/r, darunavir; ATV/r, atazanavir/ritonavir; CAH, chronic active hepatitis; HCV, hepatitis C virus; DAA, Direct-Acting Antiviral.

**Table 2:** Changes in the lipid profile during the treatment.

mg/dL	HDL	*p*	LDL	*p*	TG	*p*	Total Cholesterol	*p*
**Pretreatment**	48 ± 20		86.7 ± 34		147.7 ± 143.6		165.03 ± 46.5	
**During treatment (at 4 wks)**	47.9 ± 17	ns	105.07 ± 33.9	ns	109.4 ± 66	ns	174.8 ± 46	ns
**At the end of therapy**	51.16 ± 18.6	ns	111.4 ± 40.4	ns	110.5 ± 80.6	ns	184.7 ± 44.9	**0.0001**
**12 wks from SVR**	48.2 ± 19.2	ns	108.2 ± 43	ns	127.5 ± 93	ns	182.46 ± 52.6	**0.001**
**24 wks from SVR**	52.3 ± 21.5	ns	103.4 ± 41.38	**0.0001**	136.6 ± 108.3	ns	183.2 ± 47.3	**0.002**
**48 wks from SVR**	52.7 ± 20.2	ns	107.9 ± 41	ns	130.5 ± 123	ns	186.7 ± 48.7	**0.0001**

HDL, High-Density Lipoprotein; LDL, Low-Density Lipoprotein; TG, Triglycerides; SVR, Sustained Virologic Response; wks, weeks; ns, non-significant. The significant *p*-values are reported in bold.

## Data Availability

The raw data supporting the conclusions of this article will be made available by the authors upon request.
